# Distinct serum metabolomics profiles associated with malignant progression in the Kras^G12D^ mouse model of pancreatic ductal adenocarcinoma

**DOI:** 10.1186/1471-2164-16-S1-S1

**Published:** 2015-01-15

**Authors:** Joseph J LaConti, Evagelia C Laiakis, Anne Deslattes Mays, Ivana Peran, Sung Eun Kim, Jerry W Shay, Anna T Riegel, Albert J Fornace, Anton Wellstein

**Affiliations:** 1 Lombardi Comprehensive Cancer Center, Washington, DC 20007, United States; 2 Department of Biochemistry and Molecular & Cellular Biology, Georgetown University, Washington DC, USA; 3 UT Southwestern, Dallas, TX; 4 Center of Excellence in Genomic Medicine Research (CEGMR), King Abdulaziz University, Jeddah 22254, Saudi Arabia

## Abstract

**Background:**

Pancreatic ductal adenocarcinoma (PDAC) is the fourth leading cause of cancer deaths worldwide with less than a 6% 5-year survival rate. PDAC is associated with poor prognosis based on the late stage diagnosis of the disease. Current diagnostic tests lack the sensitivity and specificity to identify markers of early staging. Metabolomics has provided biomarkers for various diseases, stressors, and environmental exposures. In this study we utilized the p48-Cre/LSL-Kras^G12D^ mouse model with age-matched wild type mice. This model shows malignant progression to PDAC analogous to the human disease stages via early and late pancreatic intra-epithelial neoplasia (PanIN) lesions.

**Results:**

Serum was collected from mice with early PanIN lesions (at 3-5 months) and with late PanIN or invasive PDAC lesions (13-16 months), as determined by histopathology. Metabolomics analysis of the serum samples was conducted through UPLC-TOFMS (Ultra Performance Liquid Chromatography coupled to Time-of-flight Mass Spectrometry). Multivariate data analysis revealed distinct metabolic patterns in serum samples collected during malignant progression towards invasive PDAC. Animals with early or late stage lesions were distinguished from their respective controls with 82.1% and 81.5% accuracy, respectively. This also held up for randomly selected subgroups in the late stage lesion group that showed less variability between animals. One of the metabolites, citrate, was validated through tandem mass spectrometry and showed increased levels in serum with disease progression. Furthermore, serum metabolite signatures from animals with early stage lesions identified controls and animals with late stage lesions with 81.5% accuracy (p<0.01) and vice-versa with 73.2% accuracy (p<0.01).

**Conclusions:**

We conclude that metabolomics analysis of serum samples can identify the presence of early and late stage pancreatic cancer.

## Background

Identification of a circulating marker that can signal the onset of pancreatic ductal adenocarcinoma (PDAC) lesions in patients who have no outward clinical symptoms would be a great improvement with the prospect of earlier and potentially curative treatment. PDAC is the fourth leading cause of cancer death in the United States and currently has a devastating prognosis due to a <6% 5-year survival rate. Many patients initially present with advanced disease, which then limits available treatment options. Attempts have been made at establishing a method of detecting pancreatic cancer earlier through the use of biomarkers that can signal the presence of disease. The CA 19-9 assay detects a carbohydrate antigen that is elevated in patients with PDAC, but the specificity and sensitivity are not adequate for regular screening of at-risk subjects or the general population [1,2]. Other biomarkers such as Carcinoembryonic Antigen (CEA) or cell surface associated mucin 1 (MUC-1) have also shown promise in detecting pancreatic cancer by sampling the circulation, but they have suffered from the same lack of specificity and sensitivity that prevent them from being routinely recommended for patients [3,4]. Separate approaches using a global analysis of varying proteomic profiles [5-7], genomic DNA concentrations [8,9], or microRNA profiles [10] in the circulation have shown initial success in identifying the presence of PDAC in patients or in animal models, but further work has not yet been reported on the application of these assays in clinical settings.

Advances in chromatography and mass spectrometry technologies have allowed the identification of metabolites in a variety of clinical specimens. Altered metabolite concentrations can indicate aberrant enzymatic function or altered clearance mechanisms as the cause or consequence of disease. We have shown that metabolite concentration differences in biofluids such as urine and blood were effective in identifying patients with pneumonia [11], mice exposed to lipopolysaccharide [12], and as a gauge of biodosimetry during radiation treatment [13]. More studies are showing the benefits of a metabolomic approach to the identification of cancer disease states. Metabolomic analysis has offered new understanding of the pathophysioloy and diagnosis of prostate cancer [14], breast cancer [15], and colorectal cancer [16], to name a few examples.

A genetically engineered mouse model of PDAC that takes advantage of a mutated Kras protein selectively expressed in the developing pancreas has been shown in numerous studies to be an excellent model of pancreatic carcinogenesis [17]. Mice with mutant Kras^G12D^ expressed in ductal epithelia (p48-Cre/LSL-Kras^G12D^) develop early pre-malignant pancreatic intra-epithelial neoplasia (PanIN [18]) followed by progression to late PanIN (*in situ* carcinoma), invasive adenocarcinoma (PDAC) and metastasis over discrete time periods. We hypothesized that circulating metabolites in the serum of p48-Cre/LSL-Kras^G12D^ mice reveal expression profiles that could distinguish between mice with PanIN or PDAC pathology and mice with no pancreatic disease. Here we report on the overall patterns of metabolites and elevated citrate levels as one of the metabolites that was validated and shown to be related to altered expression of citrate synthase in the malignant lesions.

## Methods

### Studies in mice

All experimental procedures and animal handling were in accordance with animal protocols approved by the Georgetown University Animal Care and Use Committee (GUACUC Protocol #08-028). The *p48-Cre/LSL-Kras*^G12D^ mouse model has been described previously [17]. Briefly, a mouse strain was genetically engineered with a knockin cassette containing the *Kras* gene with a mutation coding for an amino acid change of aspartic acid to glycine at codon 12 and downstream of a stop sequence flanked by loxP excision sequences. This mouse strain was mated to another strain with the cre-recombinase enzyme under the control of the *p48* promoter that is expressed in the developing pancreas. Resulting progeny show preferential expression of the mutated Kras^G12D^ protein in pancreatic ductal epithelial cells under its endogenous promoter.

### Sample collection and analysis

Mice were housed at Georgetown University under standard 12 h light and 12 h dark cycles, and given food and water *ad libitum*. Two cohorts of male and female mice were sacrificed at either 3-5 months of age or 13-16 months of age. See Table 1 for the n of each group. Immediately before sacrifice, approximately 1 mL of blood was drawn via intra-cardiac puncture. The blood was centrifuged for 1 minute at high speed and the serum was decanted and stored at -80° C. Mice pancreata were dissected from the animals, bisected from tail to head, and fixed in formalin. A pathologist scored the highest PanIN grade per lobule of all lobules counted in a representative hematoxylin and eosin (H&E) stained slide of each mouse’s pancreas as per a similar protocol described in [17]. “Normal” includes any normal and reactive ductal change. PanIN-1 and -2 were combined into a single category of ‘early’ lesions while tissues with PanIN-3 were included in a separate category of ‘late’ lesions due to their high likelihood of malignant progression. There were three samples with invasive adenocarcinoma (PDAC) and were labeled as such.

**Table 1:** Kras^G12D^ mice of different ages have different pancreatic pathology. Shown are the numbers of mice used to measure circulating serum metabolites and the percentage of pancreatic ducts in each pathological stage.

Age Group	Pancreatic Pathology	Genoytpe	n
3-5 months	PanIN 1 and 2	Kras^G12D^	17
	normal	Wildtype	39
13-16 months	PanIN 2 and 3	Kras^G12D^	11
	normal	wildtype	16
13-16 months	PDAC	Kras^G12D^	3

Serum samples were analyzed using Ultra Performance Liquid Chromatography (UPLC) coupled to time-of-flight mass spectrometry (TOFMS) from Waters (Milford, MA). Samples were deproteinized by 1:40 dilution in 66:33 % acetonitrile:water containing 4μM debrisoquine sulfate (Sigma Aldrich, St. Louis, MO) ([M+H]^+^ = 176.1188) and 30 μM 4-Nitrobenzoic acid (Sigma Aldrich) ([M-H]^-^ = 166.0141) as internal standards. Liquid chromatography and mass spectrometry conditions were according to published material [11]. Samples were run in both positive and negative ionization modes with mass accuracy correction based on intermittent injections of sulfadimethoxine as a lockmass ([M+H]^+^ = 311.0814 and [M-H]^-^ = 309.0658). Chromatograms and ion spectra were acquired in centroid format with the software MassLynx (Waters) and data were deconvoluted with the software MarkerLynx (Waters). Significant markers were extracted with the supervised decision tree algorithm Random Forests (RF), as explained in detail in previous studies [11,12]. Putative identities of markers were acquired through online searches with the “Madison Metabolomics Consortium Database” with a 20 ppm tolerance and the “Human Metabolome Database” with a molecular weight tolerance of 0.01 Da. Validation of the putative metabolites was performed with tandem mass spectrometry against commercially available pure chemicals. Putative markers that were tested included citrate, isocitrate, uracil, uridine, AMP, phenylpyruvate, phosphorycholine, UMP, and cytidine. All chemicals were purchased from Sigma Aldrich.

### Quantification of validated markers

Quantification was performed with the triple quadrupole spectrometer ABI QTRAP 4000^®^ LC/MS/MS system (Applied Biosystems Inc, Foster City, CA) coupled to a Waters UPLC identical to the one coupled to the TOFMS. Samples were prepared in 66% acetonitrile in 1:20 dilution. All chromatographic conditions remained the same as in the discovery part. Multiple reaction monitoring (MRM) transitions were monitored on negative ionization mode for 4-NBA (*m/z* 166→122) and citrate (*m/z* 191→111). The software Analyst (Agilent Technologies, Santa Clara, CA) was used for the analysis and calculate the absolute concentrations (μM) from a standard curve of the pure chemical. Graphical representations of means ± SEM were prepared through GraphPad Prism (GraphPad Technologies, Inc.) and analysis was performed using a two-tailed Student’s T test.

### Predictions and statistical analysis

Two additional separate types of analysis were conducted with RF. A training set was developed from one third (13 wild type and 6 Kras) of the early (3-5 months) or late (13-16 months) datasets (5 wild type and 4 Kras) by randomly selecting samples and a metabolic signature was developed through RF. Based on the m/z and retention time (M_T) of the 50 metabolites (signature), their intensities were picked from the dataset for the remaining two thirds of the samples (validation set). For 3-5 months 26 wild type and 8 Kras samples were utilized, while for the 13-16 months 11 wild type and 6 Kras samples were utilized. RF was performed again and through MDS we tested how accurately this signature could classify the remainder of the samples.

A separate analysis was performed through RF, generating an early metabolomic signature (3-5 months) by combining the results from both ionization modes and applying it to the later dataset (13-16 months) for classification. The reverse analysis was also conducted to predict whether a late signature exists in the early samples for prediction of outcome. For p-value generation, non-parametric approaches were used (Chi-square test or Dixon-Mood estimates).

### Immunohistochemistry

Formalin fixed tissue specimens were sectioned and paraffin embedded. Sections were deparaffinized by overnight incubation at 60°C. Slides were submersed in xylene followed by 100%, 95%, and 70% ethanol. Antigen retrieval was performed by submerging sections in boiling citrate buffer, pH 6. Sections were washed with phosphate buffered saline (PBS) followed by quenching of endogenous peroxidase with 3% H_2_O_2_. Sections were washed and incubated in primary citrate synthase antibody (Proteintech, Chicago, IL) at dilutions recommended by the manufacturer for 1 h at room temperature in a humidified chamber. Sections were washed and incubated in rabbit conjugated –horseradish peroxidase (Dako, Denmark) diluted 1:1000 for 30 min at room temperature. DAB substrate (Dako, Denmark) was added to the sections and the reaction allowed to develop for 2-7 min. The reaction was terminated by quenching with water. Sections were counterstained with hematoxylin, dehydrated with 70%, 95%, and 100% ethanol washes and xylene submersion, followed by mounting with a glass coverslip with Drop ClearMount Solution (Zymed). All microscopy and imaging was performed using an Olympus BX40 microscope and Scion Visicapture software.

## Results

Serum was collected from p48-Cre/LSL-Kras^G12D^ mice that were 3-5 months old, 13-16 months old, and three older mice that developed PDAC along with age matched wild type controls. Table 1 describes the age groups of mice whose serum was analyzed, their genotypes, and the pancreatic pathology. The 3-5 month old mice had mostly PanIN 1 and 2 lesions, but no PanIN 3 lesions. The 13-16 month old mice had PanIN 3 lesions in ~10% of pancreatic ducts, and all control mice had a normal pancreas. The samples from different stages of progression towards PDAC reflect the state of pancreatic disease that we are most interested in identifying if they were to present in the asymptomatic state and before invasive PDAC is fully established.

### Multivariate analysis of mass spectrometry data

**Figure 1:**
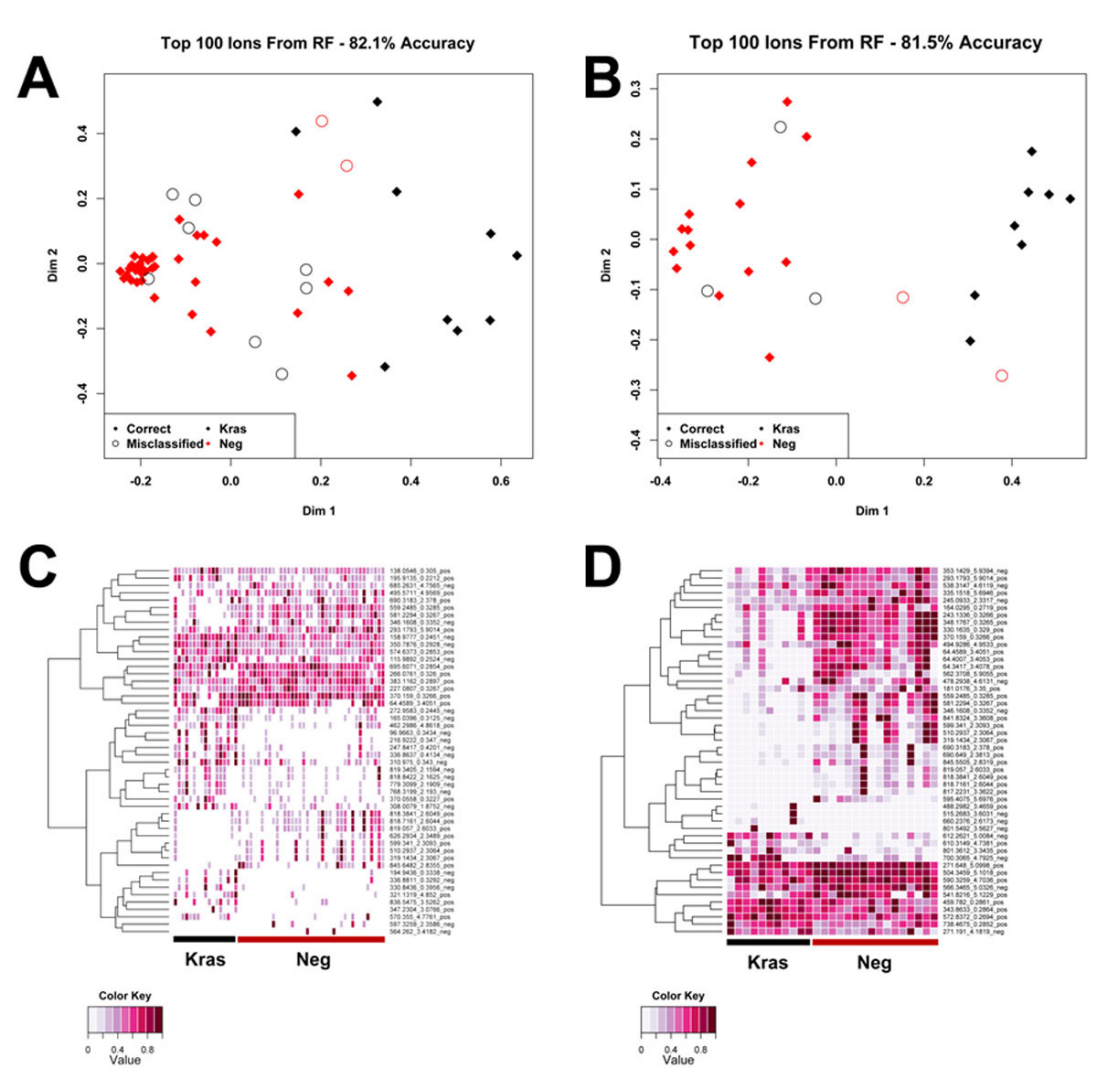
Separation of p48-Cre/LSL-Kras^G12D^ (Kras) and wild type mice based on circulating metabolite concentration profiles and heatmaps of the top 50 metabolites as classified through Random Forests (RF). Kras and wild type (neg) mice at each time point were separated based on the measurement profile of over five thousand circulating metabolites in a Random Forests multivariate data analysis. The top 100 ions, as determined through Random Forests, were used to construct a multidimensional scaling plot. Red diamonds are correctly grouped wild type mice (Neg) and open red circles are incorrectly grouped wild type mice. Black diamonds are correctly grouped Kras^G12D^ mice and open black circles incorrectly grouped Kras mice. (A) At 3-5 months the classification accuracy is 82.1 %. (B) At 13-16 months classification accuracy was 81.5 %. Heatmap displaying relative concentration differences of circulating metabolites between individual p48-Cre/LSL-Kras^G12D^ and wild type mice at 3-5 months (C) and 13-16 months (D) of age in the negative ionization mode. Black underlines Kras mice and red underlines wild type mice (Neg).

Serum samples were run on UPLC-TOFMS in both positive and negative ionization mode. Random Forests (RF) multivariate analysis was utilized to identify significant metabolites from the mass spectrometry of the serum samples. The RF analysis was concentrated on 3-5 month old p48-Cre/LSL-Kras^G12D^ (designated as Kras) mice with 3-5 month old control mice (designated as Neg), and 13-16 month old p48-Cre/LSL-Kras^G12D^ mice with their respective age matched controls. Positive and negative ionization mode data were combined to perform the analysis. The separation of groups showing the metabolic profiles of the serum samples were visualized in multidimensional scaling (MDS) plots. The accuracy of each analysis was assessed by the percentage of mice correctly assigned to the group, when including the top one hundred metabolites, as determined by RF. The metabolic profile detected in 3-5 months, separated the p48-Cre/LSL-Kras^G12D^ mice from control mice with 82.1% accuracy (Figure 1A). In the 13-16 month analysis, the percent classification accuracy was 81.5% (Figure 1B).

To illustrate the differences between the groups, the top fifty metabolites determined by RF were organized in a heatmap. The metabolites were hierarchically clustered by complete linkage using the Euclidean distance. Differential metabolic profiles were more prominent in the 13-16 month group (Figure 1D) as compared to the 3-5 month group (Figure 1C), making evident that distinct metabolomic profile differences become more prevalent as pancreatic cancer progresses.

### Validation, quantification, and immunohistochemistry

**Figure 2:**
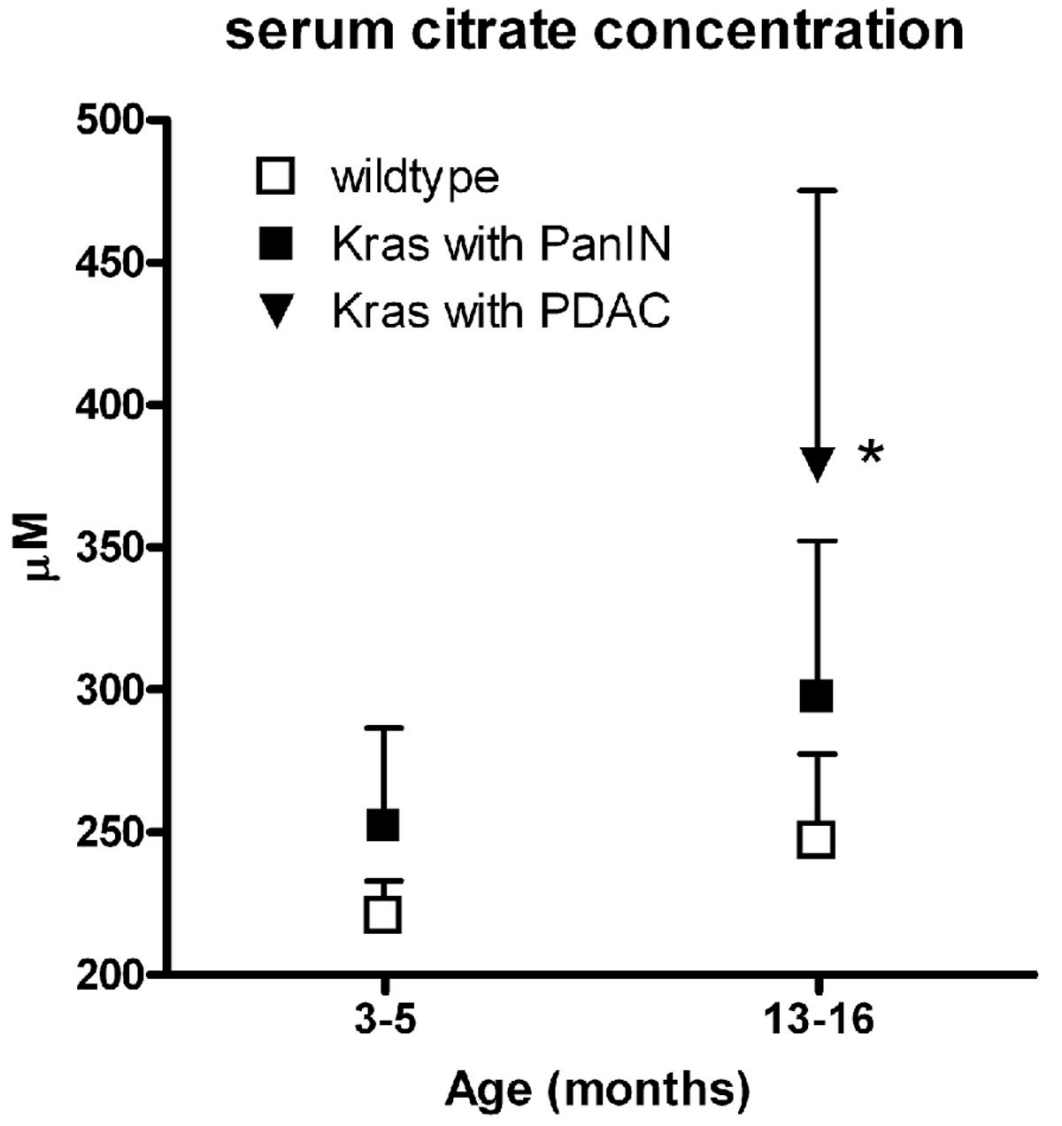
Quantification of citrate. The levels of citrate (μM citrate / μM 4-Nitrobenzoic acid) were increased in the circulation of 13-16 month old p48-Cre/LSL-Kras^G12D^ mice with PDAC compared to mice with PanIN lesions without PDAC and wild type mice. *, P<0.05 for p48-Cre/LSL-Kras^G12D^ with PDAC vs wild type at 3-5 months. Mean + SEM.

A number of metabolites identified by RF were validated through tandem mass spectrometry against pure chemicals **(see table S1, additional file**1**)**. One marker from the negative ionization mode with *m/z* of 191.0188 at a retention time of 0.4274 min was identified through online database searches as either citrate or the isobaric isocitrate. Tandem mass spectrometry revealed that the marker in question was citrate. Quantification of citrate, with the development of a standard curve (r=0.9991) using the pure chemical, revealed differential levels in the disease groups. In particular, the concentration of citrate was higher in the p48-Cre/LSL-Kras^G12D^ mice of 13-16 months of age compared to all other groups and citrate levels were found to be increased in mice with histopathological evidence of PDAC (Figure 2). Additionally, lower citrate levels were found in the serum of mice with normal pancreatic ducts when compared to mice with either PanIN or PDAC pathology.

**Figure 3:**
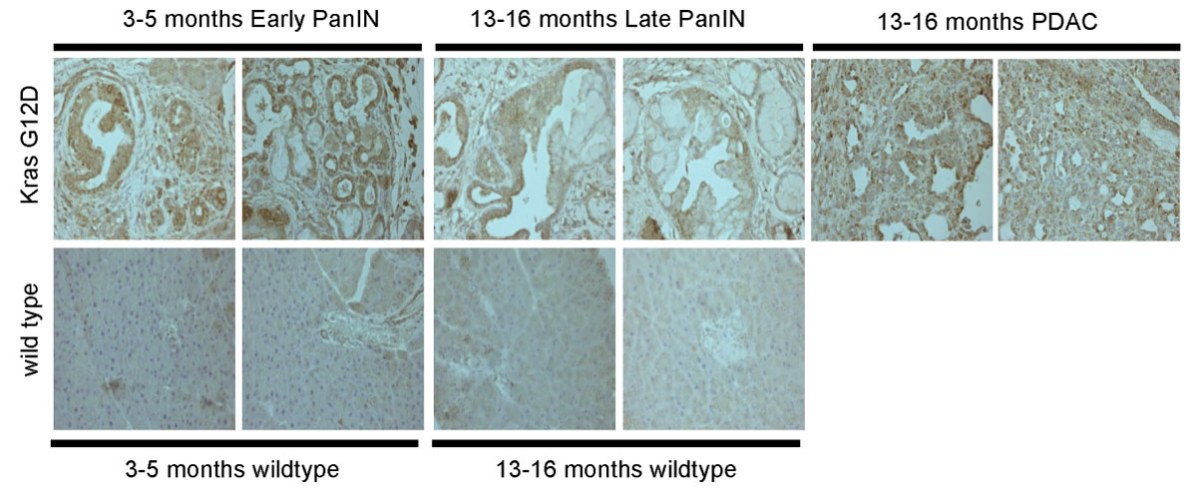
Expression of citrate synthase (CS) in immunohistochemistry. CS is detectable in PanIN ductal lesions (13-16 months old animals) and PDAC, but not wild type duct epithelia.

Immunohistochemistry of pancreatic tissue for the enzyme citrate synthase showed barely detectable synthase protein in the ducts of wild type mice and increased expression in PanIN and PDAC tissue (Figure 3). Analysis of published data with human tissue samples also showed a significant increase in expression of citrate synthase in PDAC versus normal pancreas tissues in two independent studies **(see figure S1, additional file**2**)** corroborating the findings in the preclinical model used here.

### Predictions

**Figure 4:**
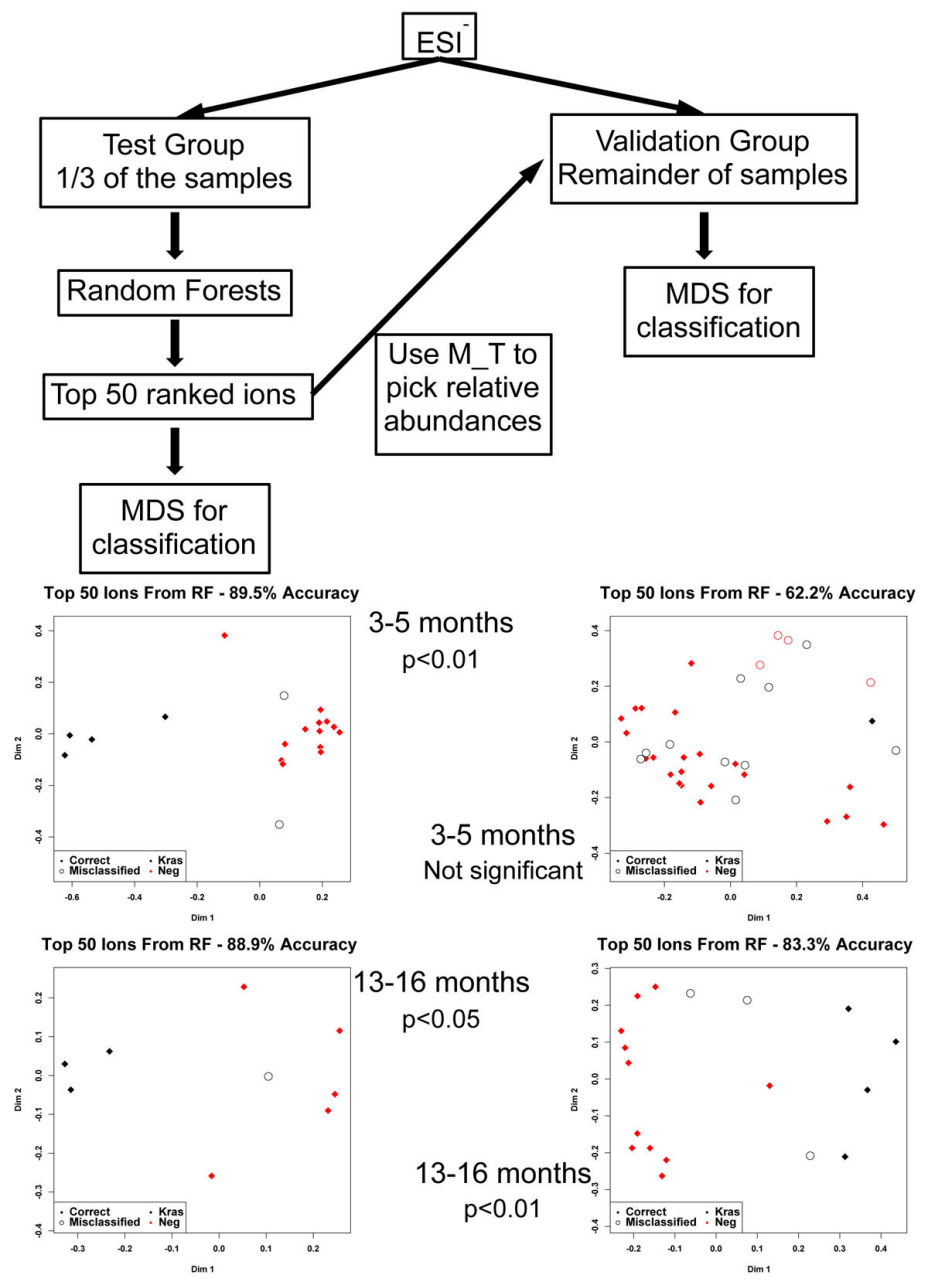
Prediction analysis based on the generation of an early or late metabolic signature. Early metabolic signatures were able to distinguish controls from diseased animals at the late stage. Supporting this, a signature from the late stage animals was able to identify animals with or without early lesions.

A metabolic signature of fifty ions was generated from the 3-5 month sample sets with combination of the positive and negative ionization data. This signature (top fifty ranked ions) was extrapolated to the 13-16 month samples for classification of the groups, as described in Figure 4. The classification accuracy of the early analysis was 82.1% (p<0.01) and at 13-16 months with extrapolation from the early time points, 81.5% (p<0.01). The reverse analysis was conducted, where a metabolic signature was created for the 13-16 month datasets (classification accuracy 85.2%, p<0.001) and extrapolated to the early time points (classification accuracy 73.2%, p<0.01).

**Figure 5:**
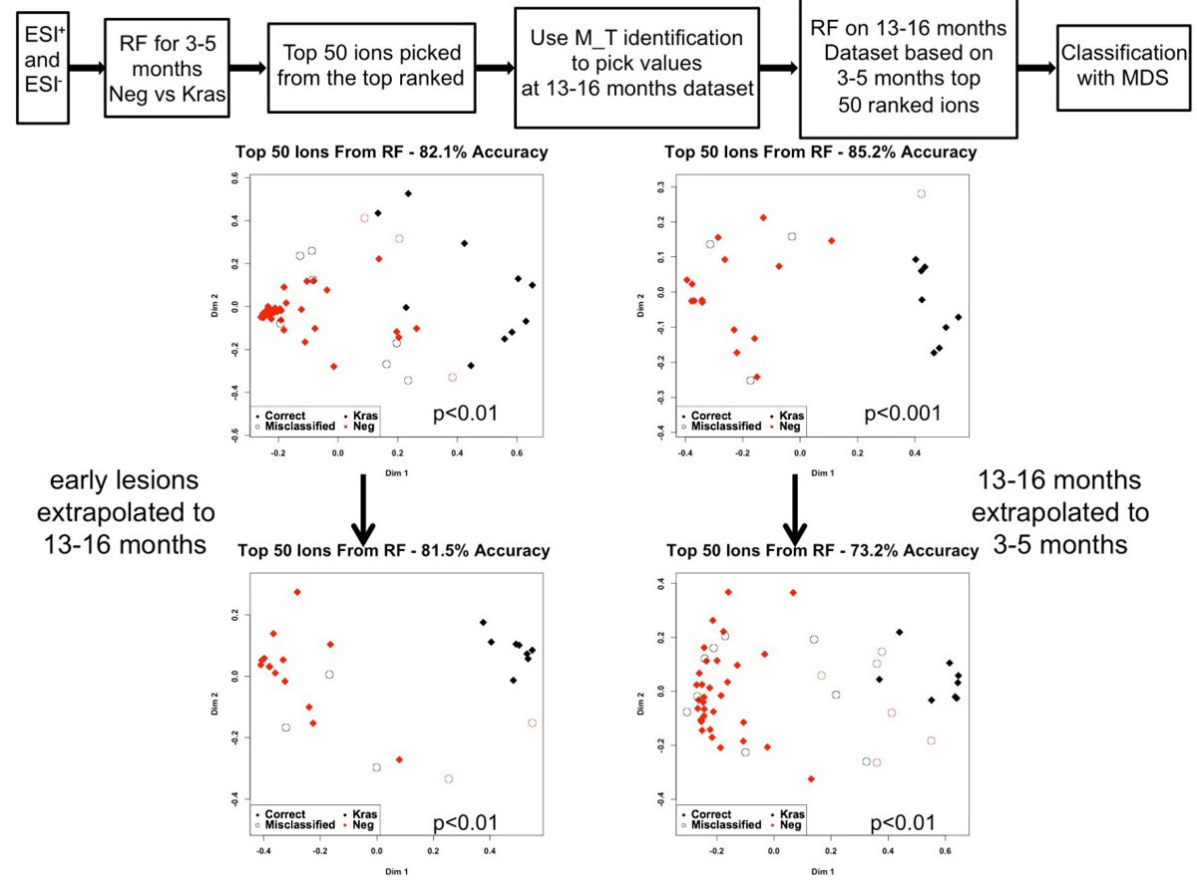
Prediction analysis based on test and validation sets. No significant predictability was observed for the stage of early lesions (3-5 months of age), whereas statistical significance was observed at the late stage (13-16 months), likely due to expression of more dominant metabolites at the later stage.

A training set (test set, one third of the samples in each group) and a validation set (the remaining two thirds of the samples) were utilized to test how stable and predictive the identified metabolic signature was across the samples (Figure 5). The classification accuracies for the training sets were 89.5% (p<0.01, two samples misclassified) for the early time points and 88.9% (p<0.05, one sample misclassified) for the late times points. The top fifty ranked ions were used from each analysis as a means of constructing a metabolic signature. This signature was utilized in the validation set to determine the stability of the metabolic changes. For the 3-5 month set classification accuracy dropped to 62.2%, with no statistical significance and fourteen samples misclassified, whereas for the later time point group classification accuracy remained high (83.3%, p<0.01, with three misclassified samples).

## Discussion

We demonstrate in this study that metabolites detected in the circulation can be used to differentiate between mice with precursor lesions of PDAC and mice with normal pancreas. More specifically, the concentration of one metabolite, citrate, was found increased in the circulation of mice with PDAC lesions compared to mice with normal pancreata, which is related to the increase in citrate synthase expression in diseased pancreatic ducts and was also seen in an analysis of data generated with human samples. Additionally, prediction analyses indicated that presence of a metabolic signature (i.e. fifty metabolites) as early as 3-5 months can be utilized as an early predictor of the cancer phenotype. Moreover, the utilization of a training and a validation set showed that diagnostic markers in a biofluid such as serum become more dominant with the progression of the disease.

There is evidence to suggest that the metabolomic profiles of patients with pancreatic cancer can be used to distinguish them from healthy subjects. Using 1H NMR and 2D NMR spectroscopy, 58 different metabolites detected in the serum of 56 pancreatic cancer patients had concentration differences that favored their separation from 43 patients with benign pancreatic lesions, **such as glutamate and acetone**[19]. Metabolites detected by 1H NMR in rat pancreatic tissue, **such as decreased phosphocholine and glycerophosphocholine**, also displayed varying concentrations that allowed the separation of rats with pancreatic cancer from healthy controls [20]. Using a similar technical approach to our own involving tandem mass spectroscopy, Urayama *et. al*. reported the separation of patients with pancreatic cancer from healthy controls based on metabolites detected in the circulation [21]. Their reported list of metabolites whose identities could be confirmed included compounds of cell membrane synthesis, aerobic respiration, and bile acid production, but did not include citrate. Wen *et. al*, found citrate levels to be increased in bile samples of patients with biliary tract cancers compared to control patients, but they did not study any cases of pancreatic cancer [22]. The evidence we present here that mice with pancreatic ductal lesions can be separated from control mice based on metabolite concentrations parallels the findings of other reports in human samples, and support the use of this genetic mouse model to test and validate future hypotheses regarding metabolomics of pancreatic tumorigenesis. This, however, is the first report of circulating citrate as a marker of pancreatic cancer progression in a mouse model, showing a connection between elevated enzyme levels in the tissue and the metabolite product in the serum. Metabolomic profiling in the serum of a pancreatic rat model has also identified increased levels of circulating citrate [23]30]vels of circulating citrate [rofiling of human K-ras oncogene transgenic rats with pancreatic ductal adenocarcinomas. ot nece. Given that current existing individual markers offer low specificity and sensitivity, it is essential to consider creating a panel of markers, i.e. signature, for early diagnosis and disease progression of PDAC. Metabolomics has the potential to contribute to this in a rapid and efficient way.

Alterations in enzymatic structure, function, or regulation can lead to a distinct change in the concentration levels of the enzyme’s substrates or products. The metabolomic analysis of biofluids for changes in metabolite concentration can imply aberrant function of a key enzymatic process. For example, isocitrate dehydrogenase 1 (IDH1) gene was found mutated in over 70% of WHO grade II and III astrocytomas and oligodendrogliomas and in glioblastomas that developed from lower grade lesions [24,25]. Metabolomic profiling of U87MG glioblastoma cells expressing these mutations in IDH1 determined the structural change of the mutated protein results in acquisition of the ability to convert α–ketoglutarate to R(-)-2-hydroxyglutarate (2HG). The authors concluded that the accumulation of 2HG commonly seen in brain tumors may be a mechanism of cellular transformation by increasing reactive oxygen species [26].

To determine if the levels of citrate were elevated in PanIN and PDAC mice as a result of increased production by citrate synthase, we examined the expression level of the enzyme in pancreatic tissue. Other reports have determined that the activity of citrate synthase was increased in pancreatic cancer cells in comparison to adjacent normal tissue [27]. Our finding that citrate synthase shows increased expression in ductal cells that have transformed to PanIN lesions provides a likely source for the increased levels of citrate found in the circulation. Analysis of mRNA levels using the Oncomine [28] database revealed increased expression in PDAC also in clinical specimens [29,30] (Figure 5) suggesting that the findings in the mouse model translate to the human disease. PanIN and PDAC transformed cells may increase their activity of citrate synthase as a result of increased cellular metabolism associated with malignant transformation and the citrate product could serve as one of the markers of the presence of incipient PDAC.

However, one biomarker may not be enough for early diagnosis of a disease. Instead, a metabolic signature, a collection of markers, needs to be developed that will be far more informative. The p48-Cre/LSL-Kras^G12D^ model that was utilized in this study shows progression from early stage lesions that resemble PanIN1/2 with a low risk of progression to invasive and metastatic cancer. On the other hand during the lifetime of the animals, high risk, late stage PanIN3 lesions as well as frank invasive and metastatic PDAC will appear. We asked whether serum metabolomic profiles of animals with low risk or early lesions would contain signatures reminiscent of late stage lesions. We also asked whether the late stage lesions contained signatures that could separate control animals from those with early stage lesions. To test these hypotheses, we generated a signature of fifty metabolites that would distinguish between control animals and those carrying early stage lesions or the respective age-matched control animals and ones with late stage lesions. It was quite striking that the serum metabolic signature in animals with early lesions was able to distinguish controls from diseased animals at the late stage. Corroborating this finding, fifty metabolites that distinguished animals with late stage lesions best were also able to identify animals with or without early lesions. We conclude from this that the serum metabolic signature at early stage of precursor lesions of PDAC contains sufficient changes to distinguish controls and late stage lesions. Future work will focus on refining this signature and generating a panel of biologically relevant markers. Still, there will be additional changes as is evident when running a cross-comparison between early and late stage lesion metabolites.

We also tested how stable the metabolic changes are across a diagnostic group. For this we randomly selected 1/3 of the respective early or late stage lesion animals and the respective controls (training set) and generated a metabolomics profile. We then applied that profile to the other 2/3 of the group and tested how well it was able to classify the respective animals (validation set). For the late stage lesions, a subgroup of 1/3 of the animals already carried the signature that was sufficient to separate controls and diseased animals in the other 2/3 of the cohort. In contrast, for early lesions no significant predictability was seen. This is likely due to less striking changes in early stage lesion animals. This analysis also indicates that with progression of the disease diagnostic metabolites become more dominant in their expression either due to the extent of the disease or the extent of changes that occur in the diseased organ. It remains to be seen whether this signature can identify animals that will progress to pancreatic cancer at an intermediate point, i.e. between 3-5 and 13-16 months.

## Conclusions

In conclusion, our work shows that distinct metabolomics profiles in serum samples as pancreatic **ductal** epithelia undergo progression towards invasive cancer and suggests that metabolomics profiling could provide a sensitive, blood-borne diagnostic signature for the presence of pancreatic cancer or its precursor lesions.

## Competing interests

The authors have declared that they have no competing interests.

## Authors’ contributions

JJL, ECL, and SEK carried out the experiments; ECL, ADM, and AW evaluated the data; JJL, ECL, ATR, AJF, and AW wrote and edited the manuscript.

## Supplementary Material

Additional file 1 **Supplementary Table 1** Putative markers tested through tandem mass spectrometry, but not validated. Putative identities were picked based on the possible biological significance through searches on online databases. Click here for file

Additional file 2 **Supplementary Figure 1** mRNA expression of citrate synthase (CS) in archival clinical tissue samples. Published cDNA arrays (Grutzmann et al [29]; Segara et al [30]) deposited in the Oncomine data base [28] and provided as median-centered, normalized data sets were downloaded and analyzed. Mean values are shown on a log2 scale + SEM. A significant increase of CS expression in PDAC versus normal pancreatic tissues is seen. Click here for file

## Declarations

This article has been published as part of *BMC Genomics* Volume 16 Supplement 1, 2015: Selected articles from the 2nd International Genomic Medical Conference (IGMC 2013): Genomics. The full contents of the supplement are available online at http://www.biomedcentral.com/bmcgenomics/supplements/16/S1
