# Supplementary figures and images for: Distinct serum metabolomics profiles associated with malignant progression in the KrasG12D mouse model of pancreatic ductal adenocarcinoma
Source: BMC Genomics. 2015 Jan 15;16(Suppl 1):S1. doi: 10.1186/1471-2164-16-S1-S1 (PMC4315147; doi:10.1186/1471-2164-16-S1-S1)

Supplementary Figure 1


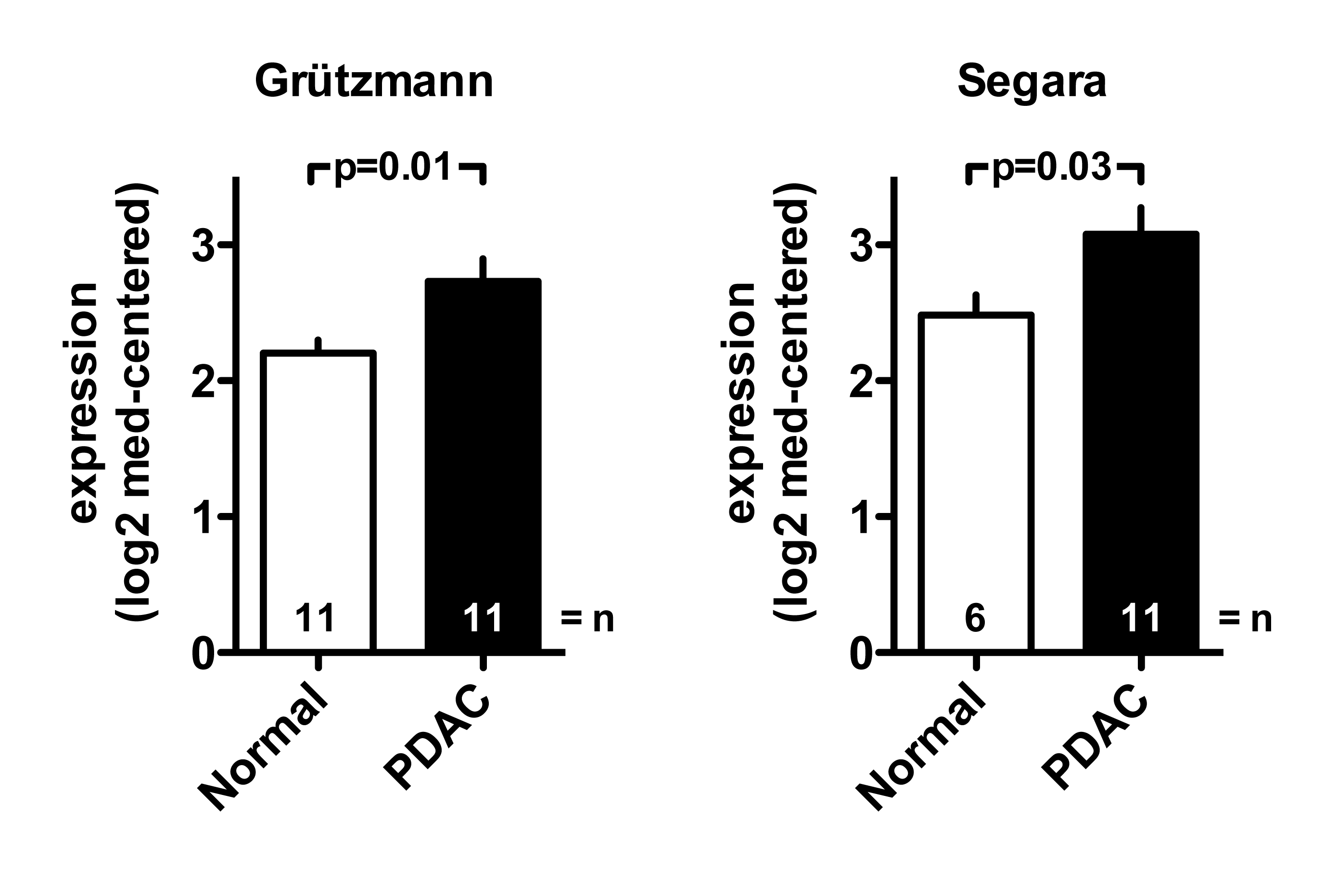

Supplement: Additional file 1 — Supplementary Table 1 Putative markers tested through tandem mass spectrometry, but not validated. Putative identities were picked based on the possible biological significance through searches on online databases. [file 1471-2164-16-S1-S1-S1.docx]
